# Tumor-associated macrophage (TAM)-derived CCL22 induces FAK addiction in esophageal squamous cell carcinoma (ESCC)

**DOI:** 10.1038/s41423-022-00903-z

**Published:** 2022-08-12

**Authors:** Jie Chen, Di Zhao, Lingyuan Zhang, Jing Zhang, Yuanfan Xiao, Qingnan Wu, Yan Wang, Qimin Zhan

**Affiliations:** 1 Key Laboratory of Carcinogenesis and Translational Research (Ministry of Education/Beijing), Laboratory of Molecular Oncology, Peking University Cancer Hospital & Institute, Beijing, 100142 China; 2 Peking University International Cancer Institute, Peking University, Beijing, 100191 China; 3 Research Unit of Molecular Cancer Research, Chinese Academy of Medical Sciences, Beijing, China; 4 Institute of Cancer Research, Shenzhen Bay Laboratory, Shenzhen, 518107 China

**Keywords:** Tumor-associated macrophages, Esophageal squamous cell carcinoma, FAK, Oncogene addiction, Cancer microenvironment, Oesophageal cancer

## Abstract

Tumor cell dependence on activated oncogenes is considered a therapeutic target, but protumorigenic microenvironment-mediated cellular addiction to specific oncogenic signaling molecules remains to be further defined. Here, we showed that tumor-associated macrophages (TAMs) produced an abundance of C-C motif chemokine 22 (CCL22), whose expression in the tumor stroma was positively associated with the level of intratumoral phospho-focal adhesion kinase (pFAK Tyr^397^), tumor metastasis and reduced patient survival. Functionally, CCL22-stimulated hyperactivation of FAK was correlated with increased malignant progression of cancer cells. CCL22-induced addiction to FAK was demonstrated by the persistent suppression of tumor progression upon FAK-specific inhibition. Mechanistically, we identified that diacylglycerol kinase α (DGKα) acted as a signaling adaptor to link the CCL22 receptor C-C motif chemokine receptor 4 (CCR4) and FAK and promoted CCL22-induced activation of the FAK/AKT pathway. CCL22/CCR4 signaling activated the intracellular Ca^2+^/phospholipase C-γ1 (PLC-γ1) axis to stimulate the phosphorylation of DGKα at a tyrosine residue (Tyr^335^) and promoted the translocation of DGKα to the plasma membrane to assemble the DGKα/FAK signalosome, which critically contributed to regulating sensitivity to FAK inhibitors in cancer cells. The identification of TAM-driven intratumoral FAK addiction provides opportunities for utilizing the tumor-promoting microenvironment to achieve striking anticancer effects.

## Introduction

The tumor microenvironment (TME) is composed of various nonmalignant stromal cells that efficiently promote tumor malignancy and significantly influence the therapeutic effectiveness of antitumor agents [1]. Various components of the TME, including stromal cells, soluble factors, metabolites, and even the extracellular matrix, can coordinately act to facilitate the malignant progression and metastasis of tumor cells. Tumor-associated macrophages (TAMs) are the most abundant immune cells and the most widely studied cellular component of the TME [2–6]. Several macrophage-related biomarkers, such as CD68, CD204, and CD206, have been used to define TAMs in the TME of ESCC [7–10]. Clinical studies have suggested a strong correlation between the TAM density and poor prognosis in patients with solid tumors, including esophageal squamous cell carcinoma (ESCC) [10–12]. Infiltration of TAMs and the tumor mutation burden are effectively predictive of the response to clinical therapies. Correspondingly, an increasing number of studies have demonstrated that TAMs can either enhance or antagonize the antitumor efficacy of cytotoxic chemotherapies and targeted or immunotherapeutic agents in specific types of solid tumors [13–15].

TAMs establish a protumor microenvironment that influences several malignant phenotypes of tumor cells, especially inducing epithelial–mesenchymal transition (EMT) and subsequently promoting the invasion or metastasis of tumor cells [16–18]. Mechanistic analyses have shown that TAMs facilitate tumor malignancy mainly by releasing various factors, such as cytokines, chemokines, and growth factors [11, 19–21]. Previous studies have identified several cytokines that are critical for TAM-mediated tumor progression, including interleukin-6 (IL-6), IL-10, CCL2, and CCL5 [19, 22–26]. The key objective of research on chemotaxis in tumor progression is to discover how chemotaxis regulates intratumoral molecular pathways and subsequently orchestrates the TME to control the metastatic spread of tumor cells. Here, we found that CCL22 (C-C motif chemokine 22), a chemokine produced by several types of immune cells, including macrophages, dendritic cells (DCs), B cells and T cells [27], was significantly upregulated in the TAMs in the ESCC microenvironment. In the TME, CCL22 mediates T_reg_ infiltration into the tumor tissue through CCR4, facilitating tumor immunosuppression and leading to inhibition of antitumor immunity [28]. However, the direct effect of TAM-derived CCL22 on intratumoral signaling pathways and the related mechanisms are still uncertain.

Several studies have reported that activated focal adhesion kinase (FAK) promotes tumor malignancy by affecting the functions of or stimulating morphological changes in tumor cells and stromal cellular components of the TME and correlates with poor prognosis in tumor patients [29, 30]. Importantly, FAK transduces signals from the extracellular microenvironment into intracellular oncogenic pathways; thus, it seems plausible that FAK might be critical for regulating the crosstalk between tumor cells and their associated microenvironment [31]. Currently, FAK inhibitors may have single-agent activity in tumors in which FAK hyperactivation or tumor cells become dependent on FAK-associated signaling [29, 30, 32]. In the present study, we aimed to investigate how TAMs mediate the hyperactivation of intratumoral FAK and whether this dysregulation of FAK has therapeutically exploitable significance.

## Results

### High expression of TAM markers correlates with poor prognosis in ESCC patients

**Fig. 1:**
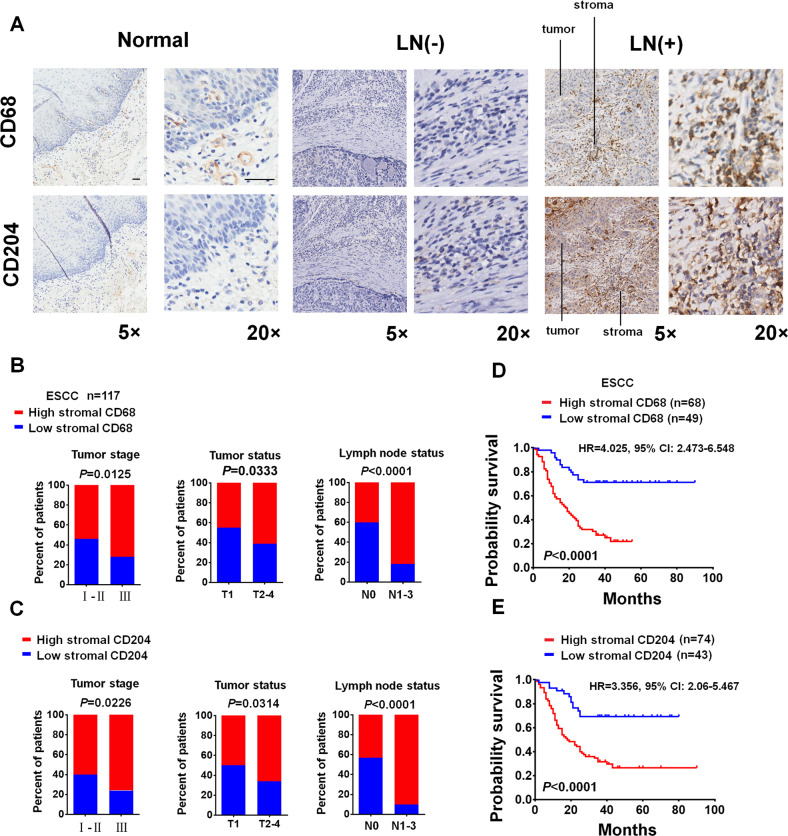
Immunohistochemical analysis of macrophages in clinical ESCC samples. **A** Representative IHC images of the expression of CD68 and CD204 in normal esophageal tissues or ESCC tissues with (LN+) or without (LN−) lymph node metastasis. **B**, **C** Percentages of ESCC patients with high expression of stromal CD68 (**B**) and CD204 (**C**) and low expression of stromal CD68 (**B**) and CD204 (**C**) according to different clinical parameters, as follows: tumor stage, tumor status and lymph node status. **D**, **E** Kaplan‒Meier curves of overall survival for ESCC patients stratified by low versus high expression of stromal CD68 (**D**) or stromal CD204 (**E**). Original magnification, ×5 or ×20

We evaluated the expression of the macrophage marker CD68 and the TAM marker CD204 in 117 ESCC tumors using an immunohistochemical (IHC) assay and found that these two markers were highly expressed in the stromal region (Fig. 1A). Importantly, increased levels of stromal CD68 and CD204 were most significantly associated with lymph node metastasis among the listed clinical parameters (Fig. 1B, C). Through Kaplan‒Meier analysis with the log-rank test, it was seen that high levels of stromal CD68 and CD204 were associated with poorer overall survival in ESCC patients (Fig. 1D, E).

### ESCC TAM-released CCL22 promotes tumor invasion

**Fig. 2:**
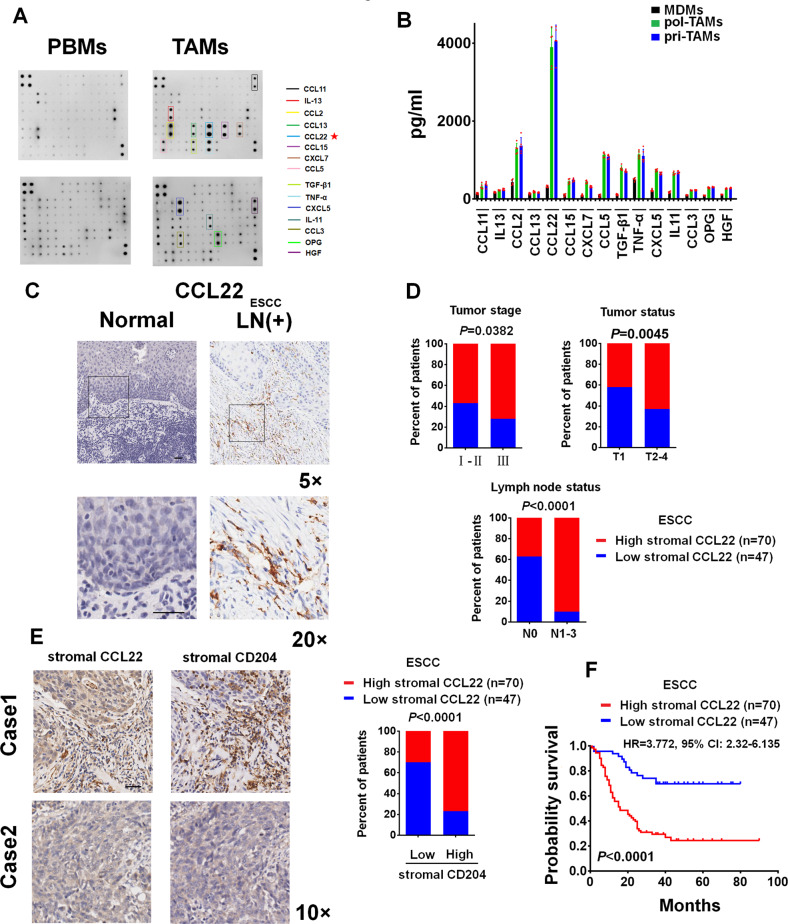
ESCC TAMs constitutively express CCL22. **A** The differential expression of cytokines in TAMs versus PBMs from the same ESCC patient, as evaluated by a cytokine antibody array assay. **B** The secretion of the indicated cytokines from MDMs, pol-TAMs, and pri-TAMs, as evaluated by ELISA. (**C**) Representative IHC images of the expression of CCL22 in normal esophageal tissues or ESCC tissues (LN+). **D** Percentages of ESCC patients with high expression of stromal CCL22 and low expression of stromal CCL22 according to different clinical parameters, as follows: tumor stage, tumor status and lymph node status. **E** Representative sequential IHC images of the expression of CD204 and CCL22 in ESCC tissues. Stromal CCL22 expression was positively associated with CD204 expression in 117 primary ESCC specimens. **F** Kaplan‒Meier curves of overall survival for ESCC patients stratified by low vs. high numbers of CCL22-positive TAMs. Original magnification, ×5, ×10 or ×20

Cytokines are important mediators of the tumor-promoting functions of TAMs [4, 33, 34]. However, the secretion profile of cytokines in ESCC TAMs is still unclear. Thus, we performed an antibody array assay to screen a panel of cytokines in paired TAMs and peripheral blood monocytes (PBMs) isolated from the same patient with ESCC. Among the cytokines tested, CCL22 was the most abundantly expressed cytokine in TAMs compared with PBMs (Fig. 2A). Then, the secretion of selected cytokines, including CCL11, IL13, CCL2, CCL13, CCL22, CCL15, CXCL7, CCL5, TGF-β1, TNF-α, CXCL5, IL11, OPG, and HGF, in primary TAMs from human ESCC tissues (pri-TAMs), polarized TAMs (pol-TAMs) from human monocyte-derived macrophages (MDMs), and MDMs was evaluated using ELISAs. CCL22 was most evidently secreted from pri-TAMs and pol-TAMs but not MDMs (Fig. 2B). The IHC data showed that CCL22-positive TAMs were primarily distributed in regions surrounding tumor cells (Fig. 2C) and were most positively associated with lymph node metastasis among the various clinical parameters (Fig. 2D). Furthermore, CCL22 was colocalized with CD204 in the stromal region in ESCC samples (Fig. 2E). Kaplan‒Meier survival analysis indicated that patients with low numbers of CCL22 TAMs survived longer than those with high numbers of CCL22 TAMs (Fig. 2F). Multiplex staining of tissues from ESCC with lymph node metastasis showed the colocalization of CD204 and CCL22 in local tumors (Figure S1A-S1C). These data strongly suggest that CCL22 is produced and secreted by TAMs and might be a biomarker for evaluating ESCC progression.

**Fig. 3:**
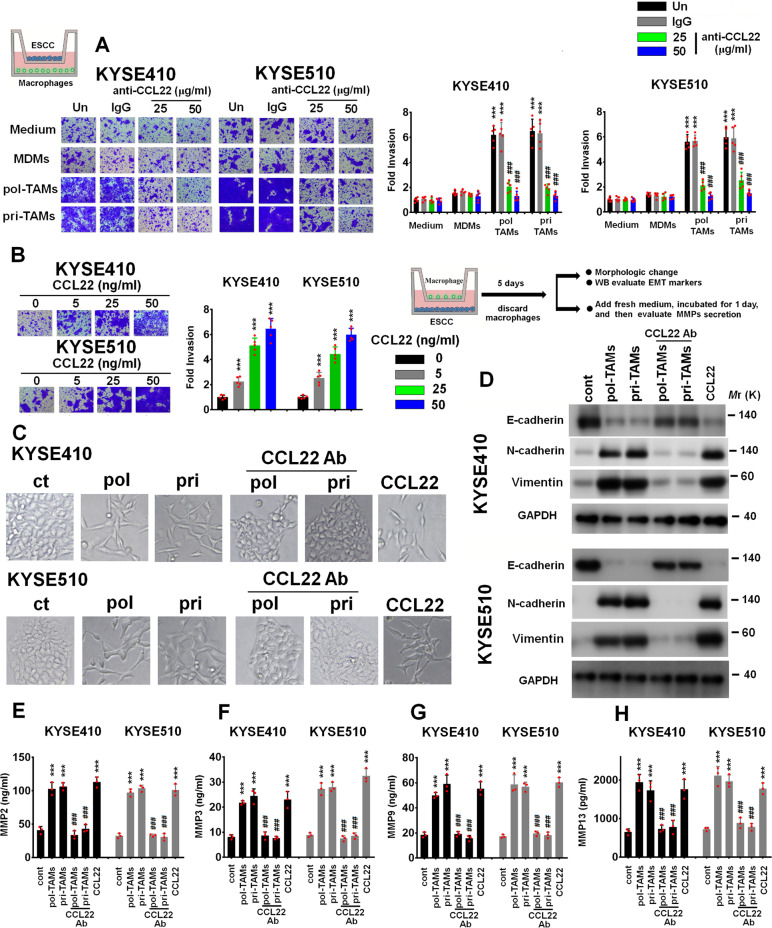
TAM-derived CCL22 promotes the invasion of ESCC cells by inducing EMT and MMP secretion. **A** Boyden chamber assay of the indicated ESCC cells plated in the upper chambers of cell culture inserts, with culture medium alone, culture medium with MDMs, culture medium with pol-TAMs, or culture medium with pri-TAMs added to the lower chambers in the presence of the anti-CCL22 antibody (25 and 50 μg/ml) or the isotype-matched IgG control (IgG). ***P < 0.001 compared with cells incubated with medium alone; ^###^*P* < 0.001 compared with untreated cells cocultured with pol-TAMs or pri-TAMs. **B** Boyden chamber assay of the indicated ESCC cells, with culture medium containing rCCL22 at increasing concentrations (5, 25, and 50 ng/ml) added to the lower chambers. ***P < 0.001 compared with control cells. (C-D) KYSE410 and KYSE510 cells (lower chamber) were cocultured with control medium, MDMs, pol-TAMs, or pri-TAMs with or without the anti-CCL22 antibody (50 μg/ml) or CCL22 (50 ng/ml) (upper chamber) in a Transwell apparatus with a 0.4 μm pore size membrane for five days. **C** Morphologic changes in the indicated ESCC cells were evaluated using phase contrast microscopy. **D** Immunoblotting was used to evaluate the expression of E-cadherin, N-cadherin, and vimentin. GAPDH was used as the internal control. **E**–**H** After coculture with the indicated macrophages in a Transwell apparatus with a 0.4 μm pore size membrane for five days, macrophages cultured in the upper chamber were discarded, and the culture medium was replaced with fresh culture medium for 1 day. The concentrations of MMP2 (**E**), MMP3 (**F**), MMP9 (**G**), and MMP13 (**H**) in the CM of the indicated ESCC cells were measured using ELISA. ***P < 0.001 compared with control cells; ^###^*P* < 0.001 compared with untreated cells cocultured with pol-TAMs or pri-TAMs

A Transwell coculture system (8 μm pore size membranes) was applied to assess the invasion of ESCC cells by plating the indicated ESCC cells in the upper chamber and macrophages in the lower chamber. Coculture with pri-TAMs and pol-TAMs effectively induced the invasion of KYSE410 and KYSE510 cells compared with the invasion of these two ESCC cell lines cultured alone. However, little increase in invasion was observed in KYSE410 and KYSE510 cells cocultured with MDMs (Fig. 3A).

We evaluated whether neutralization of CCL22 function contributes to the inhibition of TAM-mediated cancer cell invasion and found that treatment with a CCL22 neutralizing antibody (Ab) markedly decreased the number of invaded ESCC cells in cocultures with pol-TAMs or pri-TAMs. Furthermore, treatment of ESCC cells with recombinant CCL22 protein (rCCL22; 5, 25, and 50 ng/ml) for 24 h increased the invasive ability of ESCC cells in a dose-dependent manner (Fig. 3B). Two CCL22 siRNAs were transfected into pol-TAMs, and the transfection efficiency was assessed using ELISA. The CCL22 siRNAs effectively reduced the pol-TAM-induced invasion of KYSE410 and KYSE510 cells (Figure S2A and S2B).

### CCL22 induces EMT and matrix metalloproteinase (MMP) secretion in ESCC cells

EMT is the initial step of tumor invasion and metastasis in cancers, including in ESCC [35, 36]. Thus, we evaluated whether TAMs can induce EMT in ESCC cells and found that KYSE410 and KYSE510 cells cocultured with pol-TAMs, pri-TAMs or CCL22 (50 ng/ml) underwent a transition from an epithelial-like phenotype to an elongated phenotype, with a decrease in E-cadherin expression and increases in vimentin and N-cadherin expression (Fig. 3C, D). the anti-CCL22 Ab (50 μg/ml) inhibited EMT in ESCC cells induced by coculture with pol-TAMs and pri-TAMs, indicating that CCL22 is the cytokine secreted by TAMs that is responsible for EMT in ESCC cells (Fig. 3C, D).

Because EMT activates the secretion of MMPs from tumor cells to facilitate the degradation of the basement membrane and promote cell invasion [37], we further examined the secretion profiles of MMPs induced by TAM-derived CCL22 using ELISA and found that pol-TAMs, pri-TAMs, or CCL22 (50 ng/ml) effectively stimulated the secretion of MMP-2, MMP-3, MMP-9, or MMP-13 from the indicated ESCC cells. The anti-CCL22 Ab (50 μg/ml) inhibited pol-TAM- or pri-TAM-induced MMP secretion from ESCC cells (Fig. 3E–H). These results together indicate that TAM-derived CCL22 induces ESCC cell invasion by stimulating EMT and MMP secretion in tumor cells.

We further evaluated whether blockade of mesenchymal biomarkers, including N-cadherin and vimentin, or inhibition of MMP activity can suppress CCL22-mediated ESCC invasion. KYSE410 and KYSE510 cells were cocultured with CCL22 (50 ng/ml) for 5 days, and then these ESCC cells were transfected with N-cadherin or vimentin siRNAs. The transfection efficiency of the indicated siRNAs was evaluated by immunoblotting. Subsequently, these N-cadherin- or vimentin-depleted ESCC cells were subjected to an invasion assay, and the results showed that the N-cadherin and vimentin siRNAs effectively inhibited the invasion of the indicated KYSE410 and KYSE510 cells (Fig. S3A–S3C). Furthermore, KYSE410 or KYSE510 cells cocultured with CCL22 (50 ng/ml) were incubated with MMP inhibitors, including SB-3CT (25 μM; an inhibitor of MMP2 and MMP9 activity) or GM6001 (25 μM; a broad-spectrum MMP inhibitor), and then subjected to an invasion assay. As shown in Fig. S3D, these two MMP inhibitors reduced the invasive ability of CCL22-educated ESCC cells.

### CCL22-treated ESCC cells are hypersensitive to FAK inhibition

**Fig. 4:**
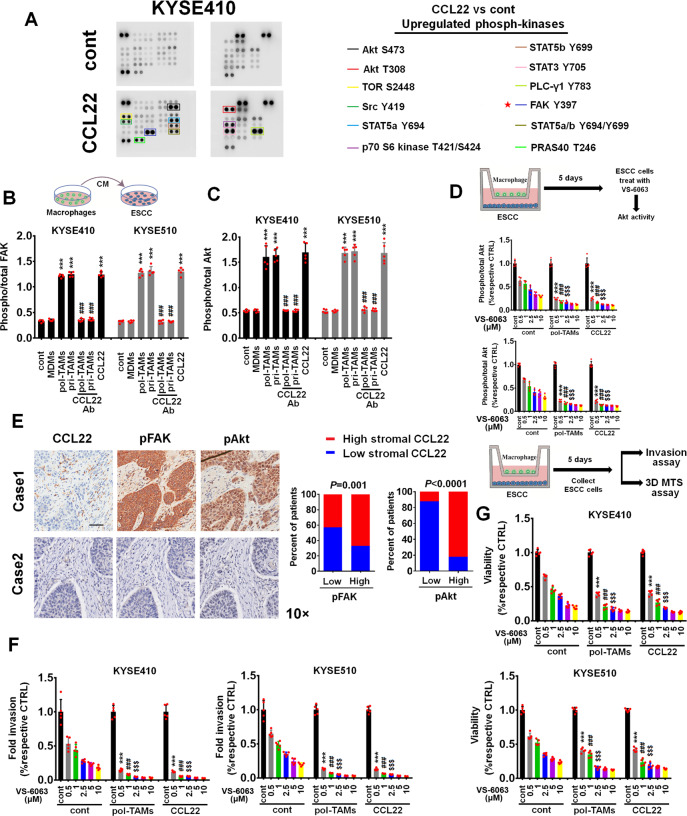
TAM-derived CCL22 activates FAK/AKT signaling in ESCC cells. **A** Total protein lysates from KYSE410 cells treated with PBS or CCL22 (50 ng/ml) were analyzed using an array of antibodies against phosphorylation sites in 43 signaling kinases (left panel). Representative sites of upregulated phosphorylation in protein kinases in CCL22-treated KYSE410 cells are listed (right panel). **B**, **C** The indicated ESCC cells were incubated with CM from MDMs, pol-TAMs, or pri-TAMs with or without the anti-CCL22 antibody (50 μg/ml) or CCL22 (50 ng/ml). The activity of FAK and AKT was assayed by a FAK Tyr^397^ (**B**) or an AKT Ser^473^ (**C**) activation quantitative ELISA. ***P < 0.001 compared with control cells; ^###^*P* < 0.001 compared with untreated cells cocultured with pol-TAMs or pri-TAMs. **D** KYSE410 or KYSE510 cells (lower chamber) were cocultured with control medium, pol-TAMs, or CCL22 (50 ng/ml) (upper chamber) in a Transwell apparatus with a 0.4 μm pore size membrane for five days and then treated with different concentrations of VS-6063 (0.5–10 μM). AKT activity was assayed by an AKT Ser^473^ activation quantitative ELISA. ***P < 0.001 compared with the indicated ESCC cells treated with 0.5 μM VS-6063 in the absence of pol-TAMs or CCL22 (50 ng/ml); ^###^*P* < 0.001 compared with the indicated ESCC cells treated with 1 μM VS-6063 in the absence of pol-TAMs or CCL22 (50 ng/ml); $$$ *P* < 0.001 compared with the indicated ESCC cells treated with 2.5 μM VS-6063 in the absence of pol-TAMs or CCL22 (50 ng/ml). **E** Stromal CCL22 expression was associated with FAK Tyr^397^ and AKT Ser^473^ phosphorylation in 25 primary human ESCC specimens. Two representative specimens with low and high levels of CCL22 are shown. Magnification, ×10 as indicated. Percentages of specimens showing low or high CCL22 expression relative to the level of pFAK or pAKT. Statistical differences were evaluated using the chi-square test. **F**, **G** KYSE410 and KYSE510 cells (lower chamber) were cocultured with control medium, pol-TAMs, or CCL22 (50 ng/ml) (upper chamber) in a Transwell apparatus with a 0.4 μm pore size membrane for five days and then treated with different concentrations of VS-6063 (0.5–10 μM). **F** The invasion ability of the indicated ESCC cells was assessed using a quantitative 96-well Boyden chamber assay. **G** The growth ability of the indicated ESCC cells was evaluated using a 3D MTS assay. ***P < 0.001 compared with the indicated ESCC cells treated with 0.5 μM VS-6063 in the absence of pol-TAMs or CCL22 (50 ng/ml); ^###^*P* < 0.001 compared with the indicated ESCC cells treated with 1 μM VS-6063 in the absence of pol-TAMs or CCL22 (50 ng/ml); $$$ *P* < 0.001 compared with the indicated ESCC cells treated with 2.5 μM VS-6063 in the absence of pol-TAMs or CCL22 (50 ng/ml)

We screened CCL22-regulated pathway kinases in ESCC cells using a phosphokinase array and found that several kinases were activated by CCL22 (50 ng/ml) in KYSE410 cells, especially those involved in FAK/AKT signaling (Fig. 4A). Quantitative ELISA showed that the phosphorylation of FAK and AKT in the indicated ESCC cells was significantly upregulated by conditioned medium (CM) from pol-TAMs or pri-TAMs and by CCL22 (50 ng/ml) but not by CM from MDMs (Fig. 4B, C). Furthermore, the anti-CCL22 Ab counteracted pol-TAM and pri-TAM CM-induced FAK and AKT activation in ESCC cells (Fig. 4B, C). We investigated whether inhibition of FAK can suppress AKT activation under stimulation with TAM-derived CCL22. Interestingly, the FAK inhibitor VS-6063 (0.5–10 μM) inhibited AKT activation in ESCC cells incubated with pol-TAMs or CCL22 (50 ng/ml) more significantly than in control ESCC cells (Fig. 4D). Clinically, the level of stromal CCL22 was positively correlated with the levels of intratumoral pFAK Tyr^397^ (*P* = 0.001) and pAKT Ser^473^ (*P* < 0.0001) in 25 ESCC tissue samples (Fig. 4E).

We further evaluated whether inhibition of FAK potentially abrogated TAM-induced malignancy in ESCC cells. The indicated ESCC cells were cocultured with CCL22 (50 ng/ml) or pol-TAMs for five days, and these ESCC cells were collected and incubated with VS-6063 (0.5–10 μM). Then, the invasion index (Fig. 4F) and proliferative ability (Fig. 4G) of these ESCC cells treated with different concentrations of VS-6063 was evaluated using a 96-well cell invasion assay and a Matrigel-based proliferation assay. Unexpectedly, the reductions in ESCC invasion (Fig. 4F) and growth (Fig. 4G) upon VS-6063 treatment were more statistically significant in ESCC cells incubated with CCL22 or pol-TAMs than in ESCC cells cultured alone.

### TAM-derived CCL22 enhances the antitumor effect of FAK inhibitor treatment in vivo

**Fig. 5:**
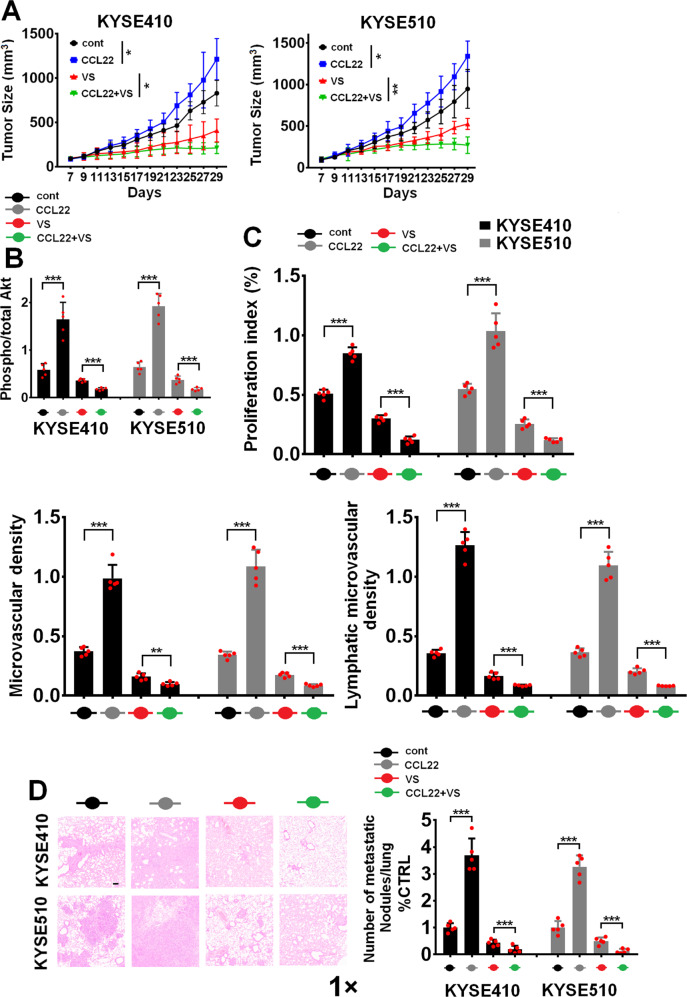
Pharmacological inhibition of FAK activity blocks CCL22-mediated ESCC malignancy in vivo. **A** The indicated ESCC cells were intravenously injected along with rCCL22 at a dosage of 0.1 μg/kg biweekly for approximately three consecutive weeks alone or with VS-6063 (25 mg/kg/day, p.o.). Tumor growth curves are shown. The ratio of pAKT/AKT in the indicated ESCC tumors from the above different groups was evaluated using ELISA. **C** The expression of Ki-67, CD31, and LYVE1 in the indicated ESCC tumor tissues was evaluated using ELISA. **D** A lung colonization model was established in mice by intravenous injection of the indicated cells via the lateral tail vein (*n* = 5 biologically independent mice per group). Representative H&E staining of lungs and the number of metastatic nodules on the surface of the lungs are shown. Magnification, ×1 as indicated. *P < 0.05, **P < 0.01, ***P < 0.001; two-tailed unpaired Student’s *t* test. Error bars, mean ± SD of five independent experiments

The hypersensitivity of TAM-stimulated ESCC cells to FAK suppression is associated with “oncogene addiction”, a biological event in which inhibition of driver oncogenes is sufficient to induce rapid and persistent tumor regression [38, 39]. We investigated whether inhibition of FAK activity in TAM-driven “FAK-addicted” cells might be therapeutically significant for blocking ESCC cell malignancy using a preclinical xenograft animal model. KYSE410 and KYSE510 cells were subcutaneously injected into the right flanks of nonobese diabetic/severe combined immunodeficiency (NOD/SCID) mice. When the xenograft volumes reached approximately 100:mm^3^, we intravenously injected CCL22 (0.1 μg/kg; twice/week) and treated the animals with VS-6063 for approximately three consecutive weeks and observed tumor growth, AKT activity, and the expression of various markers of malignant tumor progression, including Ki67 (a marker of proliferation), LYVE-1 (a marker of lymphatic vessels), and CD31 (an endothelial marker). The results in Fig. 5A, C show that intravenous injection of CCL22 into KYSE410 and KYSE510 xenografts increased the tumor size (Fig. 5A) and enhanced the activation of AKT (Fig. 5B) and the expression of Ki67, CD31, and LYVE-1 (Fig. 5C), as evaluated by quantitative ELISAs. Importantly, VS-6063 more effectively inhibited tumor malignancy in the CCL22-treated group than in the PBS-treated group (Fig. 5A, C). We compared the survival time between the different groups and found that CCL22 treatment shortened the survival time of tumor-bearing animals compared with control animals, whereas FAK inhibition caused a more statistically significant extension of survival in CCL22-treated animals than in PBS-treated animals (Figure S4A). We evaluated whether VS-6063 suppresses CCL22-induced ESCC progression using a lung colonization model. The results in Fig. 5D show that the number of tumor nests in the lungs in the CCL22 treatment group was significantly higher than that in the control group. Treatment with VS-6063 more significantly weakened the lung colonization ability of CCL22-stimulated ESCC tumors than of PBS-treated tumors (Fig. 5D).

We inoculated the indicated ESCC cells along with pol-TAMs into the flanks of NOD/SCID mice. Coinjection of pol-TAMs and tumor cells resulted in larger tumor sizes, higher AKT activity and higher Ki67, CD31, and LYVE-1 expression in tumor tissues (Fig. S5A–S5C). However, treatment with the anti-CCL22 Ab substantially reduced TAM-induced ESCC malignancy (Fig. S5A–S5C). VS-6063 significantly decreased tumor growth and the expression of the above ESCC tumor biomarkers, especially in the presence of TAMs, indicating that the FAK inhibitor exhibited a stronger antitumor effect in TAM-coinjected ESCC tumors than in non-TAM-coinjected ESCC tumors (Fig. S5A–S5C). Additionally, TAMs shortened the survival time of tumor-bearing animals (Fig. S4B), whereas the anti-CCL22 Ab was able to attenuate this effect. Importantly, FAK inhibition more effectively prolonged the survival of tumor-bearing animals in the TAM-injected group than in the non-TAM-injected group (Fig. S4B).

An in vivo lymph node metastasis model was established to evaluate the effect of TAMs on the metastatic ability of ESCC cells. The results in Figure S6 show that the lymph nodes in mice bearing tumors formed after coinoculation of pol-TAMs had larger volumes than those in mice not coinjected with pol-TAMs. In addition, VS-6063 substantially decreased the volume of lymph nodes in the ESCC cell/pol-TAM coinjection group compared with the group injected with ESCC cells alone (Fig. S6). Collectively, our results suggest that TAM-derived CCL22 induces hypersensitivity to FAK inhibition due to the addiction to hyperactive FAK.

### CCL22 induces FAK activation via CCR4/PLC-γ1 axis-mediated DGKα membrane recruitment

**Fig. 6:**
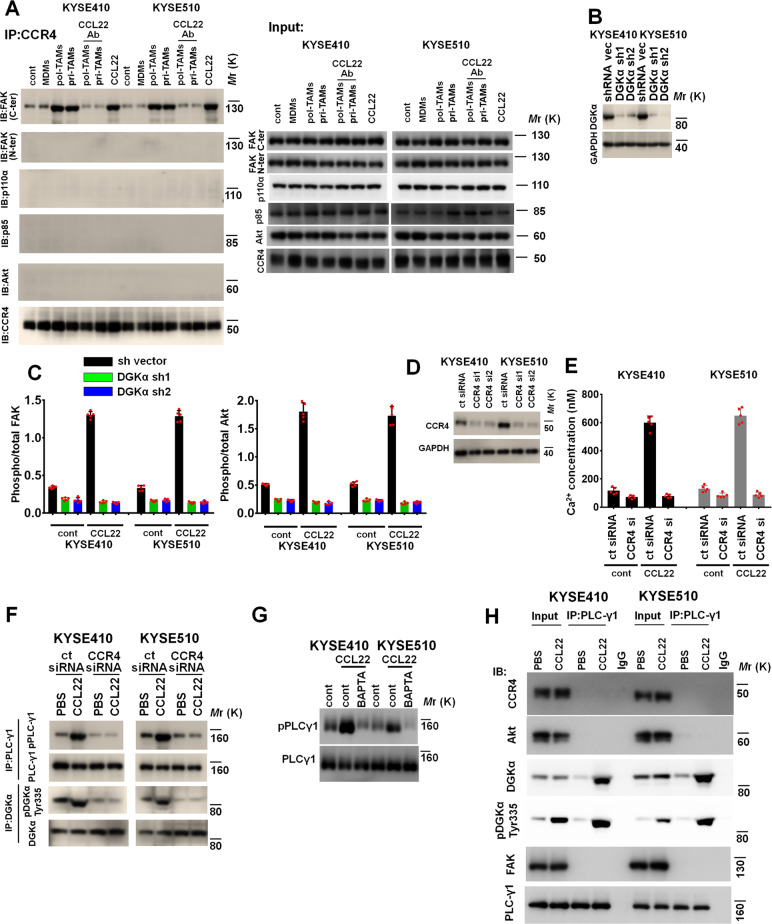
CCL22 induces the assembly of the CCR4/DGKα/FAK complex in ESCC cells. **A** Immunoprecipitation-immunoblotting showed that CCR4 interacted with only the FAK C-terminal and not the FAK N-terminal, PI3K catalytic subunit p110α, PI3K regulatory subunit p85, or AKT in KYSE410 and KYSE510 cells with the indicated treatment. **B** Silencing of DGKα in ESCC cells transduced with the two indicated short hairpin (sh) RNAs was analyzed by immunoblotting. GAPDH was used as the loading control. **C** The indicated control shRNA- and DGKα shRNA-transduced ESCC cells were treated with PBS or CCL22 (50 ng/ml). The activity of FAK and AKT was assayed by a FAK Tyr^397^ (left panel) or an AKT Ser^473^ (right panel) activation quantitative ELISA. **D**–**F** Control and CCR4-depleted ESCC cells were treated with or without CCL22 (50 ng/ml). **D** Silencing of CCR4 in ESCC cells transfected with the two indicated small interfering (si) RNAs was analyzed by immunoblotting. GAPDH was used as the loading control. **E** The Intracellular Ca^2+^ concentration was evaluated using a calcium detection assay kit. **F** Protein lysates were immunoprecipitated with an anti-PLC-γ1 or anti-DGKα antibody and then subjected to immunoblotting to evaluate the protein levels of pPLC-γ1 Tyr^783^ and pDGKα Tyr^335^. **G** KYSE410 and KYSE510 cells were pretreated with the Ca^2+^ chelator BAPTA-AM (10 μM) for 90 minutes and then incubated with CCL22 (50 ng/ml). Lysates were collected to evaluate the expression of pPLC-γ1 Tyr^783^ and PLC-γ1 using immunoblotting. **H** Immunoprecipitation-immunoblotting showed that PLC-γ1 interacted with DGKα but not FAK, CCR4, or AKT in KYSE410 or KYSE510 cells treated with CCL22 (50 ng/ml)

Because CCR4 is the receptor for CCL22 [27], we further evaluated whether intratumoral CCR4 is critical for mediating CCL22-induced FAK activation. The immunoprecipitation assay showed that CM from pol-TAMs, CM from pri-TAMs, and CCL22 (50 ng/ml) induced a physical interaction between CCR4 and the FAK C-terminus but not the FAK N-terminus, PI3K p85α, p110α, or AKT, whereas the anti-CCL22 Ab (50 μg/ml) disrupted the pol-TAM- or pri-TAM-induced interaction between CCR4 and the FAK C-terminus (Fig. 6A).

Although CCL22 stimulates the CCR4/FAK axis, there is no evidence that CCR4 has a direct effect on the phosphorylation of FAK at Tyr^397^ (the phosphorylation site for FAK-mediated PI3K/AKT activation in the N-terminus of FAK). We thus speculated that at least one other factor mediates CCL22-induced FAK Tyr^397^ phosphorylation. DGKα has been proposed to directly interact with the N-terminus of FAK to mediate the phosphorylation of FAK at Tyr^397^ [40]. We depleted DGKα in KYSE410 and KYSE510 cells using shRNA and then treated these ESCC cells with CCL22 (50 ng/ml) to observe the activation of intratumoral FAK and AKT (Figs. 6B, C). The ELISA results showed that DGKα shRNA markedly inhibited CCL22-mediated FAK/AKT activation in ESCC cells (Figs. 6B, C).

**Fig. 7:**
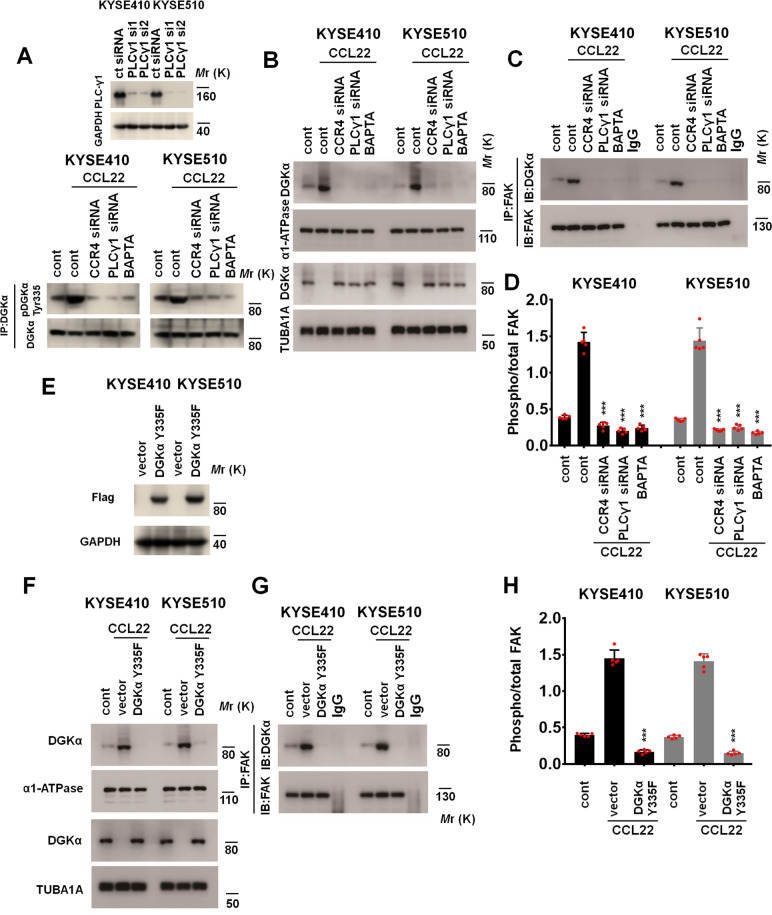
CCL22 induces membrane recruitment of DGKα via the Ca^2+^/PLC-γ1 axis. **A** Silencing of PLC-γ1 in ESCC cells transfected with the two indicated siRNAs was analyzed by immunoblotting. GAPDH was used as the loading control (upper panel). Control or CCR4-treated PLC-γ1 siRNA-transfected KYSE410 and KYSE510 cells were pretreated with the Ca^2+^ chelator BAPTA-AM (10 μM) for 90 min and then incubated with CCL22 (50 ng/ml). Protein lysates were immunoprecipitated with an anti-DGKα antibody and then subjected to immunoblotting to assess the level of pDGKα Tyr^335^. **B**–**D** Control or CCR4-treated PLC-γ1 siRNA-transfected KYSE410 and KYSE510 cells were pretreated with the Ca^2+^ chelator BAPTA-AM (10 μM) for 90 min and then incubated with CCL22 (50 ng/ml). **B** Cell membrane and cytosolic proteins were extracted, and immunoblotting was used to detect the expression of DGKα, α1-ATPase (a membrane marker) and TUBA1A (a cytoplasmic marker). **C** Immunoprecipitation-immunoblotting was used to evaluate the interaction between DGKα and FAK at the plasma membrane. **D** FAK activity was assessed by ELISA. **E** The transfection efficiency of the DGKα Y335F plasmid in the indicated ESCC cells was evaluated. GAPDH was used as the loading control. **F**–**H** Vector or DGKα Y335F mutant KYSE410 and KYSE510 cells were treated with CCL22 (50 ng/ml). **F** Cell membrane and cytosolic proteins were extracted, and immunoblotting was used to detect the expression of DGKα, α1-ATPase (a membrane marker) and TUBA1A (a cytoplasmic marker). **G** Immunoprecipitation-immunoblotting was used to evaluate the interaction between DGKα and FAK at the plasma membrane. **H** FAK activity was assessed by ELISA. ***P < 0.001, two-tailed unpaired Student’s *t* test. Error bars, mean ± SD of five independent experiments

We assessed the concentration of intracellular calcium ([Ca^2+^]_*i*_) upon CCL22 treatment using a calcium detection assay, which has been extensively applied to evaluate the activation of chemokine receptors [41]. The results in Fig. 6D, E show that CCL22 (50 ng/ml) induced an increase in the intracellular Ca^2+^ concentration in control ESCC cells but not in CCR4 siRNA-transfected ESCC cells. Correspondingly, the results of immunoblotting showed that CCL22 (50 ng/ml) induced the phosphorylation of PLC-γ1, which is known to be a mediator in the Ca^2+^_*i*_ pathway (Fig. 6F). However, transfection of CCR4 siRNA inhibited CCL22-induced phosphorylation of PLC-γ1 (Fig. 6F). The Ca^2+^ chelator BAPTA-AM (10 μM) effectively inhibited CCL22-induced phosphorylation of PLC-γ1 in KYSE410 and KYSE510 cells (Fig. 6G). A previous study found that PLC-γ1 directly phosphorylated Tyr^335^ of DGKα in an in vitro system, and activation via phosphorylation of this site is critical for membrane recruitment of DGKα [42]. The results in Fig. 6H show that PLC-γ1 interacted with DGKα but not with CCR4, FAK or AKT in the indicated ESCC cells treated with CCL22 (50 ng/ml). Furthermore, the change in the DGKα Tyr^335^ phosphorylation status was positively correlated with the change in the PLC-γ1 phosphorylation status in the indicated ESCC cells (Fig. 6F). Blockade of the CCR4/Ca^2+^/PLC-γ1 axis with the Ca^2+^ chelator BAPTA-AM (10 μM), CCR4 siRNA or PLC-γ1 siRNA effectively inhibited CCL22-mediated phosphorylation of DGKα at Tyr^335^ (Fig. 7A). Stable depletion of intratumoral PLC-γ1 by shRNA effectively inhibited CCL22-induced lymphatic metastasis of ESCC cells in vivo (Fig. S7A and S7B).

Disruption of the CCR4/Ca^2+^/PLC-γ1 axis effectively blocked CCL22-induced membrane recruitment of DGKα (Fig. 7B), the interaction between DGKα and FAK (Fig. 7C), and FAK activation (Fig. 7D). After the catalytically inactive DGKα Y335F mutant was transfected into ESCC cells (Fig. 7E), the CCL22-facilitated membrane recruitment of DGKα, the DGKα/FAK interaction, and subsequent FAK activation were blocked (Fig. 7F–H). We further evaluated whether inhibition of DGKα/FAK activity can reduce the secretion of MMPs from ESCC cells; to this end, we treated CCL22-treated KYSE410 and KYSE510 cells with VS-6063 (2.5 μM). After FAK inhibition, the secretion of MMP2, MMP3, MMP9, and MMP13 from CCL22-treated KYSE410 and KYSE510 cells was significantly decreased (Fig. S8A–S8D). Similarly, expression of the DGKα Y335F mutant effectively inhibited CCL22-induced MMP secretion from KYSE410 and KYSE510 cells (Fig. S8E–S8H). These observations strongly suggest that the CCL22/CCR4 axis is the driving force for the direct interaction between FAK and DGKα and for FAK activation in tumor cells.

## Discussion

The interaction between TAMs and tumor cells is pivotal for tumor malignancy [22, 43, 44]. In this study, we demonstrated that the majority of macrophages localized in the peritumoral regions of ESCC exhibited a polarized phenotype. Importantly, TAMs could be used as an effective predictor for evaluating the malignancy and survival of patients with ESCC. We also found that CCL22 was highly expressed in ESCC TAMs and correlated with the malignant progression of ESCC. CCL22 drives the recruitment of T_reg_ cells to the tumor tissue and contributes to an immunosuppressive microenvironment, thus potentially impairing antitumor immunity. Our in vitro coculture system experiments and xenograft animal model revealed that TAM-derived CCL22 directly induced ESCC cell invasion and lymphatic metastasis by inducing EMT and promoting MMP secretion from tumor cells, expanding the tumor-promoting effect of CCL22 on ESCC. Through combined clinical analyses and preclinical functional assays, we demonstrated that TAM-secreted CCL22 could be used as an effective diagnostic or prognostic predictor of ESCC invasion. However, future investigations on the dynamic interactions between ESCC cells and TAMs or several types of T cells at both the cellular and molecular levels are critical to amplify our understanding of how TAMs function in controlling tumor malignancy and the TME, which may help to facilitate the development of therapeutic strategies for ESCC.

TAM-derived CCL22 has been shown to be overexpressed in several solid tumors [45–47]. However, the direct stimulatory effect of CCL22 on intratumoral signaling and tumor malignancy is less understood. We found that CCL22 increased the phosphorylation of various intratumoral signaling substrates, especially FAK, the crucial hub that transduces tumor microenvironmental signals into tumor cells [30, 48, 49] and regulates multiple malignant phenotypes during tumor progression. Oncogene addiction arises when cancer cells become reliant on a single oncogenic pathway to induce tumor malignancy and has traditionally been thought of as an intrinsic characteristic of tumor cells [50, 51]. Functionally, pharmacological inhibition of FAK activity significantly inhibited the TAM-mediated enhancement of ESCC malignancy, endowing the TME/tumor ecosystem with profound hypersensitivity to FAK inhibition. Importantly, the translational significance of the present study is that harmful TAM-derived CCL22 might unexpectedly amplify the antitumor effect of FAK inhibitors, suggesting a novel approach to target TAM-induced oncogene addiction. A previous study showed that the FAK inhibitor VS-4718 effectively inhibited the progression of tumors with low expression of E-cadherin, a characteristic of EMT [52]. Combining this study and our results, we hypothesized that EMT in ESCC cells resulted in increased cell motility and was associated with metastasis, which was accompanied by an increased dependency on FAK activation and, therefore, increased sensitivity to FAK inhibition. Various strategies to deplete TAMs or neutralize their mediators have been used to reduce tumor malignancy in preclinical tumor models. However, the efficacy of these strategies is still controversial [13–15, 53]. The CCL22-mediated FAK addiction in ESCC cells implies that TME-derived cytokines can prime the responsiveness of cancer cells to protein tyrosine kinase-based targeted therapies by inducing oncogene addiction and indicates that an immunosuppressive TME may be the critical switch to mediate oncogene addiction.

Although CCL22 stimulates the interaction between CCR4 and FAK, no evidence shows the direct stimulatory effect of CCR4 on the phosphorylation of FAK at Tyr^397^. DGKα was found to directly interact with the FAK FERM domain to release the inhibitory effect of the N-terminus on FAK and subsequently stimulate phosphorylation of FAK at Tyr^397^. Here, we found that DGKα functions as a signaling adaptor to mediate the CCR4/FAK pathway. Depletion of DGKα effectively blocked CCL22/CCR4 chemotaxis-induced FAK/AKT activation. Our mechanistic studies showed that CCL22-activated CCR4 could effectively mediate the membrane recruitment of DGKα via a series of biological events, including mobilization of Ca^2+^_*i*_ and activation of PLC-γ1 (an important mediator in Ca^2+^ signaling pathways), to ultimately phosphorylate a tyrosine residue (Tyr^335^) in DGKα, after which DGKα interacts with membrane-localized CCR4 in ESCC cells. Combined with the result that depletion of CCR4 blocks the interaction between DGKα and FAK, our data indicated that the CCL22-initiated assembly of the CCR4/DGKα/FAK protein complex may facilitate the DGKα-mediated increase in FAK phosphorylation in response to stress imposed by TAMs.

Although Ca^2+^-activated PLC-γ1 was the main effector of the CCL22/CCR4 axis in membrane recruitment of DGKα in the present study, Ca^2+^ functioned as the second messenger, with a broad profile of effectors [54, 55], which may induce membrane recruitment of DGKα via other mechanisms. Our finding of the phosphorylation-dependent adaptor function of DGKα revealed an important mechanism by which the CCR4/DGKα/FAK complex coordinately regulates CCL22-induced tumor metastasis. Combined with the observation that DGKα promotes tumor metastasis by directly activating the FAK/AKT pathway [40], the results of the present study augmented the evidence that the DGKα/FAK axis contributes to ESCC progression in the context of the TME.

**Fig. 8:**
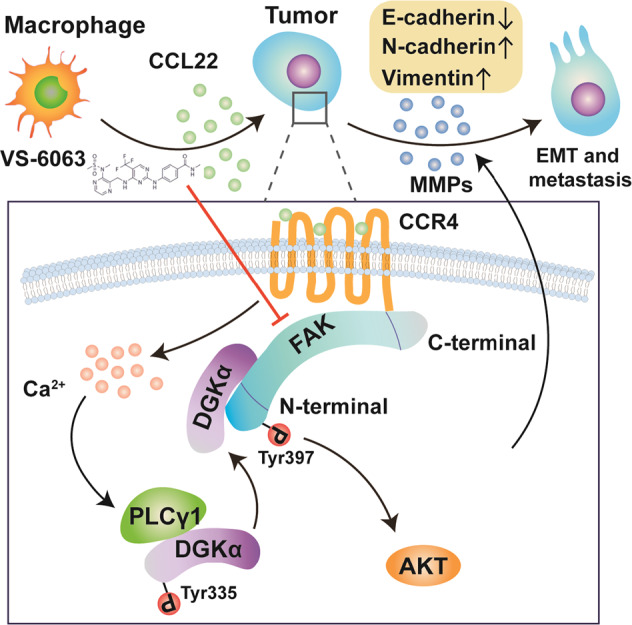
Proposed model of TAM-derived CCL22-induced oncogenic addiction to hyperactivated FAK. TAM-derived CCL22 facilitates the assembly of the CCR4/DGKα/FAK complex to stimulate the constitutive hyperactivation of FAK. This series of molecular events induces tumor cells to become hypersensitive to FAK inhibition and promotes EMT and MMP secretion in ESCC cells

The lack of effective treatment strategies for metastatic ESCC has strongly driven scientists and clinicians to urgently explore novel pharmacological targets and identify relevant molecular mechanisms for tumor intervention. Importantly, the success of FAK inhibition in the present study highlights the translational potential of establishing FAK as a target for ESCC treatment in the setting of crosstalk between tumor cells and the TME (Fig. 8).

## Methods

### Cell culture and transfection

PBMs from patients with ESCC were obtained by density gradient centrifugation using Ficoll-Hypaque (Pharmacia), and pri-TAMs were collected from freshly removed tumor tissues with a Percoll Density Gradient Centrifugation Kit (Pharmacia). Then, monocytes (PBMs) and macrophages were isolated with CD14 MicroBeads (Miltenyi) according to the manufacturer’s instructions [56, 57]. The origin and culture conditions of the ESCC cell lines KYSE410 and KYSE510 were as described in our previous study [40].

For siRNA transfection, the indicated cells were plated at 5 × 10^5^ cells/ml in serum-free medium and transfected with specific siRNA duplexes and Lipofectamine 2000 (Invitrogen). The oligonucleotide sequences of the siRNAs were as follows: CCR4 siRNA1, 5′-GGACCTTGCAGCATTGTAA-3′; siRNA2, 5′-GGTCTGTGCAAGATGATTT-3′. PLC-β1 siRNA1, 5′-GCTTCTATGTAGAGGCAAA-3′; siRNA2, 5′-GCAAGAAGTTCCTTCAGTA-3′. N-cadherin siRNA1, 5′-CCAATCAACTTGCCAGAAA-3′; siRNA2, 5′-GTGCAACAGTATACGTTAA-3′. Vimentin siRNA1, 5′-GGCACGTCTTGACCTTGAA-3′; siRNA2, 5′-CCTTGAACGCAAAGTGGAA-3′. CCL22 siRNA1, 5′-GCGTGGTGAAACACTTCTA-3′; siRNA2, 5′-GCCGTATTACGTCCGTTA-3′.

Short hairpin RNA (shRNA) was applied to stably deplete the expression of DGKα or PLC-γ1 in the indicated cells, and the sequences of the DGKα shRNAs and the transfection protocols were as described in our previous study [40]. The shRNA sequences of PLC-γ1 were as follows: PLC-γ1 shRNA1, 5′-GGAATCGTGAGGATCGTATAT-3′; shRNA2, 5′-GCCATTGACATTCGTGAAATT-3′.

For mutant transfection experiments, pcDNA3.1-Flag-DGKα Y335F was transfected into the indicated cells for 48 hours. Cell lysates were collected for immunoprecipitation (IP) assays.

### Production of pol-TAMs

PBMs were cultured in DMEM supplemented with 10% fetal bovine serum and M-CSF (10 ng/ml) to generate MDMs [58, 59]. To generate pol-TAMs, MDMs were cultured with IL-4 (20 ng/ml) [57–59]. Then, pol-TAMs were collected and cultured in 1 mL of RPMI 1640 medium in the lower chamber of a 24-well Transwell apparatus for 24 h and subsequently subjected to other assays.

For production of conditioned medium (CM) from pol-TAMs, after generating pol-TAMs, M-CSF and IL-4 were removed, and pol-TAMs were further cultured with fresh RPMI 1640 medium (FBS-free) for another 24 h. CM was collected for further studies.

### Clinical ESCC samples and immunohistochemical analysis

Clinical ESCC samples were collected with approval from the institutional review board of Peking University Cancer Hospital. For immunohistochemical staining, deparaffinized and rehydrated slides were boiled in Tris-EDTA buffer (10 mM) for 15 minutes for antigen retrieval. The slides were then incubated with antibodies against CD68 (1:500), CD204 (1:500), CCL22 (1:100), pFAK Tyr^397^ (1:500) and pAKT Ser^473^ (1:500) overnight at 4 °C. 3,3′-Diaminobenzidene tetrahydrochloride (DAB; Dako) was applied to detect the indicated antibodies. The levels of CD68, CD204, and CCL22 were quantitatively calculated using the following formula: staining score = SI (staining intensity, which was defined as follows: 0 = negative; 1 = weak; 2 = moderate; 3 = strong) × PP (percentage of positive cells, which was defined as follows: 0 = 0%; 1 = 0–25%; 2 = 25–50%; 3 = 50–75%; 4 = 75–100%). For evaluation of the CD68, CD204, and CCL22 expression, the cutoff values for low and high expression were ≤3 and >3, respectively.

### Xenograft model

KYSE410 and KYSE510 cells were subcutaneously injected into the flanks of NOD/SCID mice (female; five weeks old; Vital River Laboratory Animal Technology Co., Ltd.). When the xenograft volumes reached approximately 100 mm^3^, intravenous injection of PBS and rCCL22 (0.1 μg/kg; twice/week) was conducted for approximately three weeks (*n* = 5 mice/group). The animals in the CCL22 (0.1 μg/kg, twice/week, i.v.) treatment group were also treated with VS-6063 (25 mg/kg/day, p.o.). Tumor dimensions were measured using a digital caliper, and tumor volumes were calculated according to the following formula: volume (mm^3^) = (length (L) × width (W)^2^) × 0.5. In the lung colonization model, KYSE410 and KYSE510 cells were intravenously injected into animals (*n* = 5 mice/group), and the animals were then intravenously injected with PBS or CCL22 (0.1 μg/kg, twice/week, i.v.) and treated with VS-6063. The number of metastatic tumor nodules in the lungs was determined using hematoxylin and eosin (HE) staining according to standard protocols.

For the coinjection mouse model, KYSE410 or KYSE510 cells and pol-TAMs were coinoculated into the flanks of animals in 0.1 ml of sterile PBS. When the xenograft volumes reached approximately 100 mm^3^, the animals were treated with the indicated agents (*n* = 5 mice/group). The treatment period lasted approximately three weeks.

The effect of pol-TAMs and CCL22 on the lymphatic metastasis of ESCC cells was assessed in an animal model of popliteal lymph node metastasis. The exact experimental procedures were described in a previous study [40].

### Statistical analysis

GraphPad 7.0 was applied to perform all statistical analyses. To evaluate differences between two independent groups in most of the in vitro and animal experiments, two-tailed unpaired Student’s *t* test was performed. For clinical sample analyses, the chi-square test and the Kaplan‒Meier method were used as indicated. The error bars in the graphs of experimental data indicate the standard deviation (SD) of three to five independent experiments. A *P* value of <0.05 was considered statistically significant.

Other materials and methods are described in the Supplementary methods.

## Supplementary information

Supplementary Figures 1-8
