# Supplementary material for: Tumor-associated macrophage (TAM)-derived CCL22 induces FAK addiction in esophageal squamous cell carcinoma (ESCC)
Source: Cell Mol Immunol. 2022 Aug 12;19(9):1054–66. doi: 10.1038/s41423-022-00903-z (PMC9424285; doi:10.1038/s41423-022-00903-z)
Supplement: Supplementary file 1 — Supplementary Figures 1-8 [file 41423_2022_903_MOESM1_ESM.doc]

**Supplementary materials and methods:**

**Reagents and antibodies:**

Antibodies against DGKα (Cat# H00001606-B01P), CD204 (Cat# PAB30252), and CD68 (Cat# MAB12126) were from Abnova. Antibodies against CCL22 (Cat# ab9847), CCR4 (Cat# ab83250), α1-ATPase (Cat# ab7671), and TUBA1A (Cat# ab200216) were from Abcam. E-cadherin (Cat# 3195), N-cadherin (Cat# 13116), Vimentin (Cat# 5741), PI3K p110α (Cat# 4255), p85 (Cat# 4292), AKT (Cat# 9272), PLC-γ1 (Cat# 2822), pPLC-γ1 (Tyr^783^) (Cat# 14008), pFAK Tyr^397^ (Cat# 3283S), Flag (Cat# 2368), and GAPDH (Cat# 5174) were from CST. Antibody against FAK (N-terminal) (Cat# AM2249b) was from Abcepta. pDGKα (Tyr^335^) was synthesized by ABclonal. Human CCL22 neutralizing antibody (Cat# MAB336) and recombinant human CCL22 protein (Cat# 336-MD/CF) were from R&D systems. pDGKα (Tyr^335^) antibody was synthesized by Abclonal. FAK inhibitor (VS-6063) (Cat# S7654), SB-3CT (Cat # S7430), and GM6001 (Cat # S7157) were from Selleck Chemicals.

**Transwell invasion assay**

Invasion of ESCC cells was examined using 24-well Boyden chambers (Corning, NY) with 8 μM-inserts coated with and Matrigel. Indicated ESCC cells were cultured in the upper chambers at 37 ºC. The lower chamber was incubated with the indicated macrophages. After 24 hours, the invaded cells that crossed the inserts were stained with 2% crystal violet (Sigma), and were counted as cells per field of view under phase-contrast microscopy.

For evaluating the anti-invasive ability of VS-6063 on tumor cells, indicated tumor cells were incubated with pol-TAMs or CCL22 (50 ng/ml) for 5 days, and then tumor cells were collected to assess the anti-invasive ability of VS-6063 using CytoSelect 96-well cell invasion assay with basement membrane (Cell Biolabs; Cat# CBA-112) according to the manufacturer’s instructions.

For assessing whether blockade of EMT affected the invasion of ESCC cells, KYSE410 or KYSE510 cells were incubated with CCL22 (50 ng/ml) for 5 days. Then, these ESCC cells were collected to incubate with N-cadherin or vimentin siRNAs, and these N-cadherin or vimentin-depleted ESCC cells were subjected to invasion assay for 24 hours. For measuring whether neutralization of MMPs affected the invasion of ESCC cells, KYSE410 or KYSE510 cells were incubated with CCL22 (50 ng/ml) for 5 days, and then these ESCC cells were treated with SB-3CT (25 μM), or GM6001 (25 μM) and subjected to invasion assay for 24 hours. The invasive ability of ESCC cells was evaluated using EZCell^TM^ cell invasion assay kit (Biovision; Cat # K913), and the exact protocols were according to the manufacturer’s instructions.

**3D cell culture MTS assay**

Before MTS assay, indicated tumor cells were incubated with pol-TAMs or CCL22 (50 ng/ml) for 5 days. Then, Indicated cells were collected and suspended in 2% Matrigel-containing media and seeded in the 96-well plates with the indicated doses of VS-6063 for 4 days. MTS assay was then used to evaluate cell viability and to assess the tumor inhibitory effect of VS-6063 on tumor cells.

**Immunoprecipitation (IP) assay**

Cells were lysed with IP lysis buffer (25 mM Tris-HCl (pH 7.4), 150 mM NaCl, 1% NP-40, 1 mM EDTA, 5% glycerol and protease inhibitors), and centrifuged at 10, 000 for 15 min at 4 °C. Supernatants were incubated with first antibody (diluted at 1:100) overnight at 4 °C. Then, the lyses were incubated with Pierce^TM^ protein A/G agarose (Thermo; Cat#20421) for 2 hr at 4 °C. Immunocomplexes were then washed 5 times with lysis buffer and boiled in SDS-loading buffer, and then subjected to immunoblotting with indicated antibodies.

For membrane protein extraction, Mem-PER^TM^ plus membrane protein extraction kit (Thermo; Cat# 89842) was used, and the exact protocol was according to manufacturer's instruction. The extraction efficiency of membranous components was evaluated using immunoblotting to detect the expression of α1-ATPase (membranous marker; 1:1000) and TUBA1A (cytoplasmic marker; 1:1000). Cellular membranous extracts were incubated with appropriate primary antibodies (diluted at 1:100), and then lyses were incubated with Pierce^TM^ protein A/G agarose (Thermo; Cat#20421) for 2 hours at 4 °C.

**ELISA**

Human CCL11/Eotaxin ELISA Kit (R&D systems; Cat# DTX00), human IL13 ELISA kit (R&D systems; Cat# D1300B), human CCL2/MCP-1 ELISA kit (R&D systems; Cat# DCP00), human CCL13/MCP4 ELISA kit (Raybiotech; Cat# ELH-MCP4), human CCL22/MDC ELISA kit (R&D systems; Cat# DMD00), human CCL15/MIP1 delta ELISA kit (Raybiotech; Cat# ELH-MIP1d), human CXCL7/NAP2 ELISA kit (Raybiotech; Cat# ELH-NAP2), human CCL5/RANTES ELISA kit (R&D systems; Cat# DRN00B), human TGFβ1 ELISA kit (R&D systems; Cat# DB100B), human TNFα ELISA kit (Raybiotech; Cat# ELH-TNFa), human CXCL5/ENA78 ELISA kit (R&D systems; Cat# DX000), human IL11 ELISA kit (R&D systems; Cat# D1100), human CCL3/MIP1α (R&D systems; Cat# DMA00), human Osteoprotegerin (OPG) ELISA kit (Raybiotech; Cat# ELH-OPG), and human HGF ELISA kit (R&D systems; Cat# DHG00B), were applied to measure the secretion of these indicated cytokines from MDMs, pol-TAMs, or pri-TAMs. The exact experimental protocol was according to manufacturer’s instructions.

For measurement of activated FAK (pFAK Tyr^397^/FAK ratio) and AKT (pAKT Ser^473^/AKT ratio), cells with or without the indicated treatment were cultured in 12-well plates at 80% confluence. Levels of activated FAK and AKT in the cell tumor lysates from the indicated groups were measured using the human phospho-FAK (Tyr^397^) ELISA Kit (Raybiotech; Cat# PEL-FAK-Y397; with minor modification to evaluate the level of total FAK), and the human phospho-AKT (Ser^473^) ELISA Kit (Raybiotech; Cat# PEL-AKT-S473-T) following the manufacture's protocol. The activation status of FAK or AKT was evaluated according to the formula that the optical density (OD) value of pFAK or pAKT/the OD value of total FAK or AKT.

For evaluation of MMPs secretion from the indicated ESCC cells, levels of MMP2, 3, 9, or 13 in the cell supernatants were measured using the human MMP2 ELISA Kit (Raybiotech; Cat# ELH-MMP2-1), human MMP3 ELISA Kit (Raybiotech; Cat# ELH-MMP3-1), human MMP9 ELISA Kit (Raybiotech; Cat# ELH-MMP9-1), human MMP13 ELISA Kit (Raybiotech; Cat# ELH-MMP13-1) according to the manufacturer's instructions. For evaluation of tumor-promoting biomarkers in indicated tumor tissues, human Ki-67 ELISA kit (Raybiotech, Cat# ELH-MKI67-1), human PECAM-1 (CD31) ELISA kit (Raybiotech, Cat# ELH-PECAM1-1), or human LYVE-1 ELISA kit (Raybiotech, Cat# ELH-LYVE1-1), was used following manufacturer’s instructions.

**Antibody arrays**

For comparing the different cytokines between paired PBMs and TAMs, lysates were added to Human cytokine antibody arrays against 120 unique cytokines (Raybiotech; catalog AAH-CYT-1000) and processed according to the manufacturer's instruction. Briefly, arrays were blocked, incubated with approximate 500 μl of lysates overnight, followed by biotin-conjugated antibodies (1:250) incubation for 2 hours and with HRP-linked secondary antibody (1:1000) for 1 hr. For the phospho-kinase activation study, antibody arrays against 43 kinase phosphorylation sites (R&D systems; catalog# ARY003) were used according the manufacturer's instruction. Cell lysates (approximate 500 μl) were added to each membrane. The membranes were incubated with chemiluminescent substrate and exposed for 10 minutes.

**Intracellular Ca^2+^ evaluation**

For evaluation of intracellular Ca^2+^ level, cells were cultured in 6-well plates at 80% confluence and grown under the indicated stimulation for 1 hour. Then, cells were harvested and suspended in 500 μl calcium assay buffer and put on ice for quickly pipetting a few times, and subsequently centrifuged at 15000 *g* at 4 °C for 10 minutes to obtain supernatants. Ca^2+^ level in the supernatants was evaluated using the Calcium detection assay kit (Abcam; Cat# ab102505) according to the manufacturer's instruction. The experiment was repeated five times.

**Multiplex immunofluorescence**

Clinical ESCC samples (5 μm thick; formalin-fixed and paraffin-embedded, FFPE) were deparaffinized in xylene, rehydrated, and washed before boiling in Tris-EDTA buffer for antigen retrieval. 10% normal goat serum was used to block nonspecific binding for 30 minutes. The primary antibodies against CD204 (1:500), CCL22 (1:100) were incubated overnight at 4 °C. DAPI was used to stain cell nucleus. The signals were visualized using 3Dhistech Pannoramic Scan system.

**Supplementary Figures and Figure legends:**


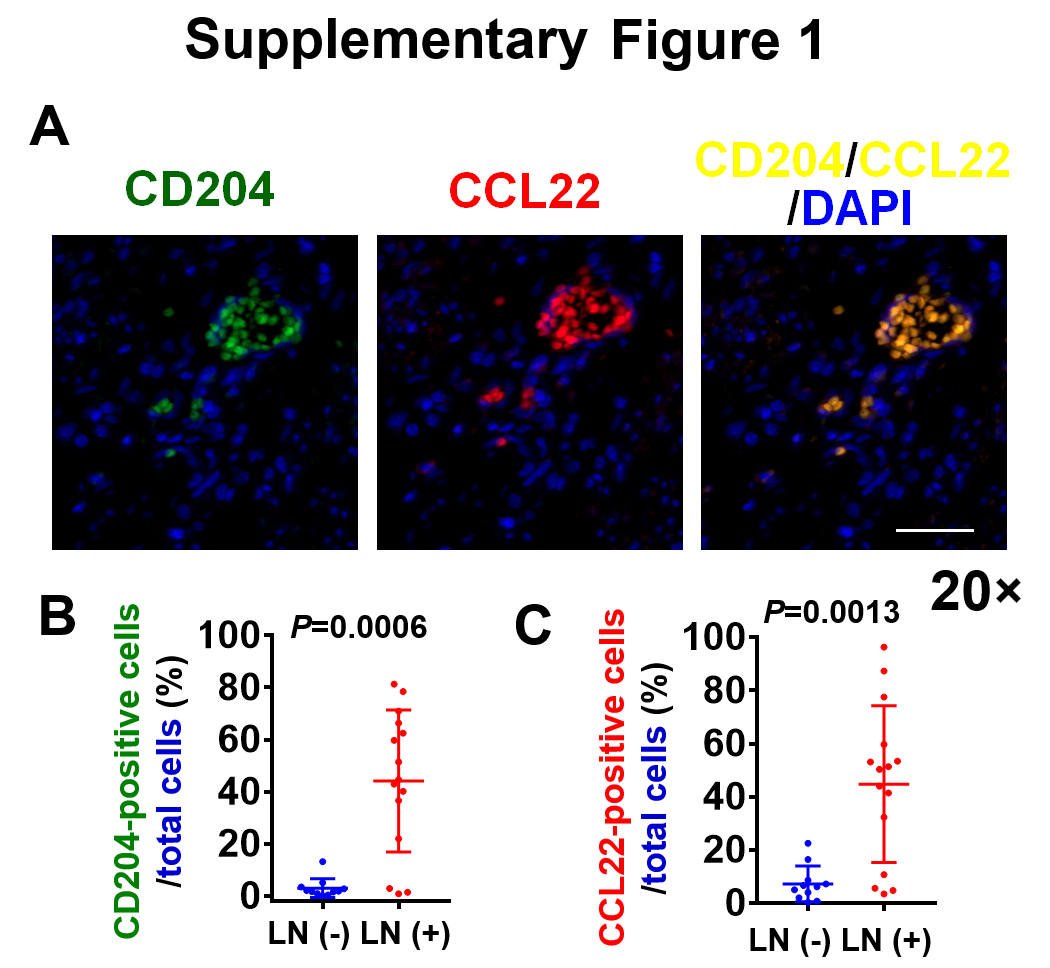


Supplementary Figure 1:

1. Representative images of CD204 and CCL22-positive TAMs surrounding ESCC with lymph node metastasis stained by multiplex IHC staining. Original magnification, 20 ×. (B-C) Statistical analyses of the expression of CD204 (B) and CCL22 (C) in LN (-) and LN (+) ESCC tissues. Mann-Whitney U test. *P* value was shown.

**
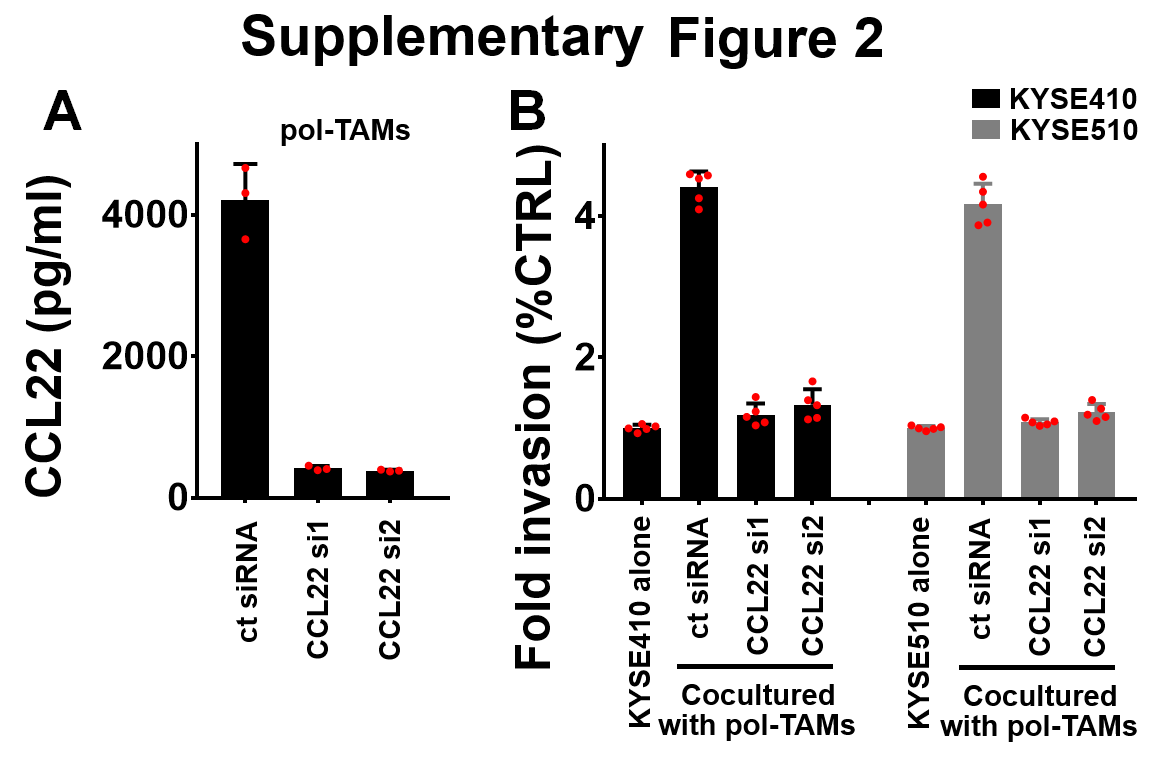
**

Supplementary Figure 2:

1. Pol-TAMs were transfected with two CCL22 siRNAs, and the knockdown efficacy was evaluated using ELISA assay to measure the CCL22 secretion from pol-TAMs. (B) KYSE410 or KYSE510 cells were plated on the upper inserts of transwell apparatus (8 μm pore size), and the control siRNA or CCL22 siRNAs were plated in the lower chambers. The invasive ability of indicated ESCC cells were evaluated using EZCell^TM^ cell invasion assay.

**
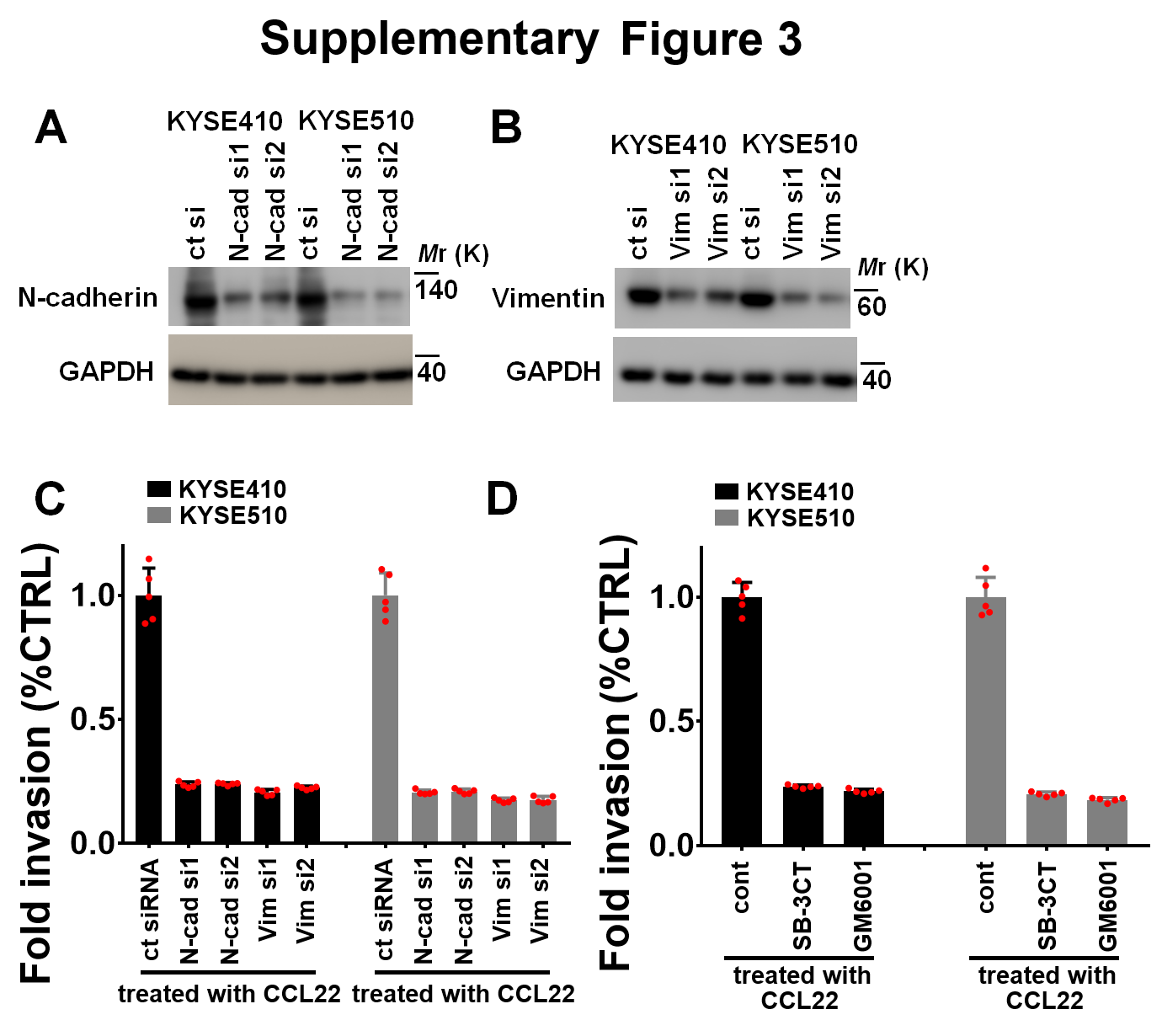
**

Supplementary Figure 3:

(A-B) KYSE410 or KYSE510 cells were cocultured with CCL22 (50 ng/ml) for 5 days. Then, KYSE410 or KYSE510 cells were respectively transfected with N-cadherin (A) or vimentin (B) siRNAs. The transfection efficacy was evaluated using immunoblotting assay. GAPDH was served as internal control. (C) Indicated ESCC cells harbored N-cadherin or vimentin siRNAs were plated on the upper inserts of transwell apparatus (8 μm pore size) for 24 hours, and the invasive ability of indicated ESCC cells were evaluated using EZCell^TM^ cell invasion assay. (D) KYSE410 or KYSE510 cells were cocultured with CCL22 (50 ng/ml) for 5 days. Then, KYSE410 or KYSE510 cells were collected and plated on the upper inserts of transwell apparatus (8 μm pore size), and treated with SB-3CT (25 μM), or GM6001 (25 μM) for 24 hours. The invasive ability of indicated ESCC cells were evaluated using EZCell^TM^ cell invasion assay.

**
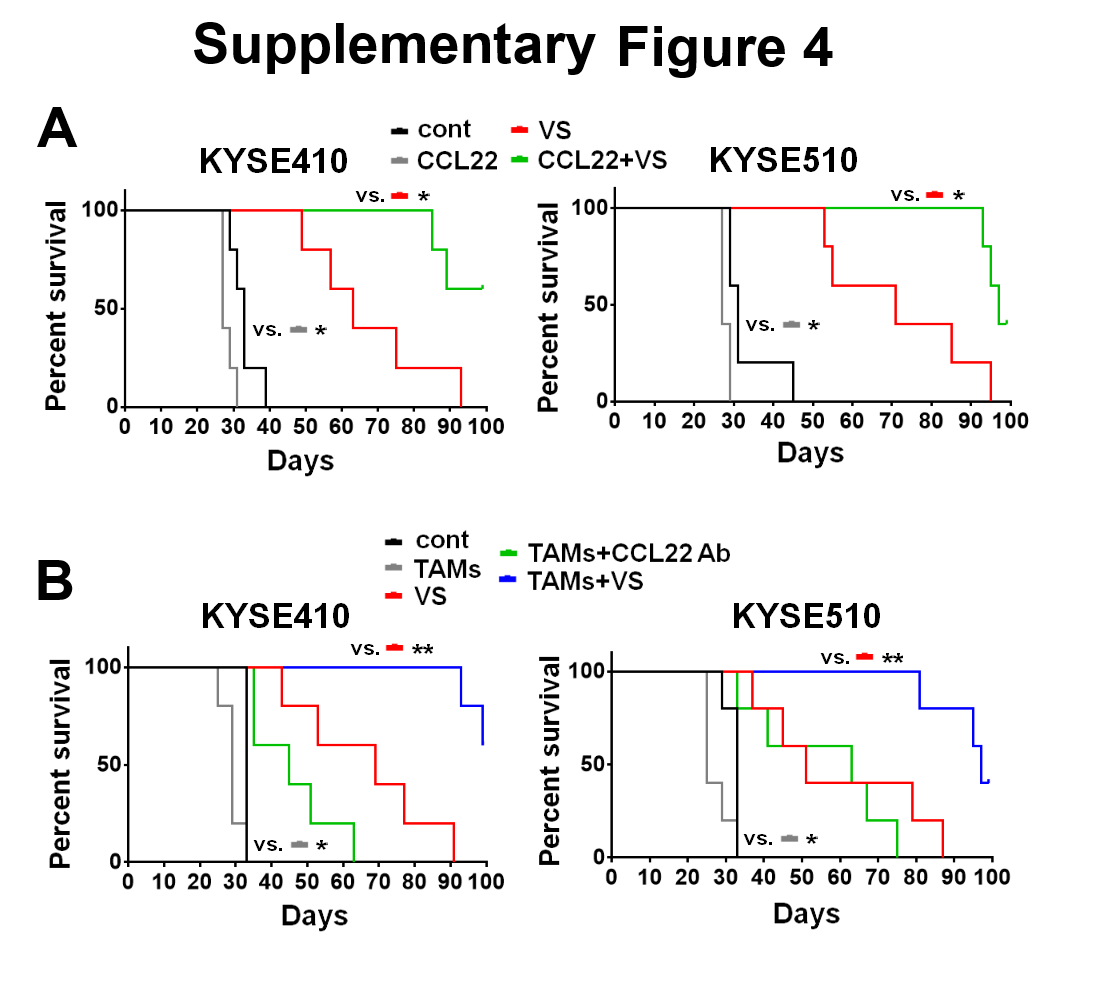
**

Supplementary Figure 4:

1. B) Kaplan-Meier curves for illustration of the survival periods of xenografted mice with indicated treatments (n=5/group).


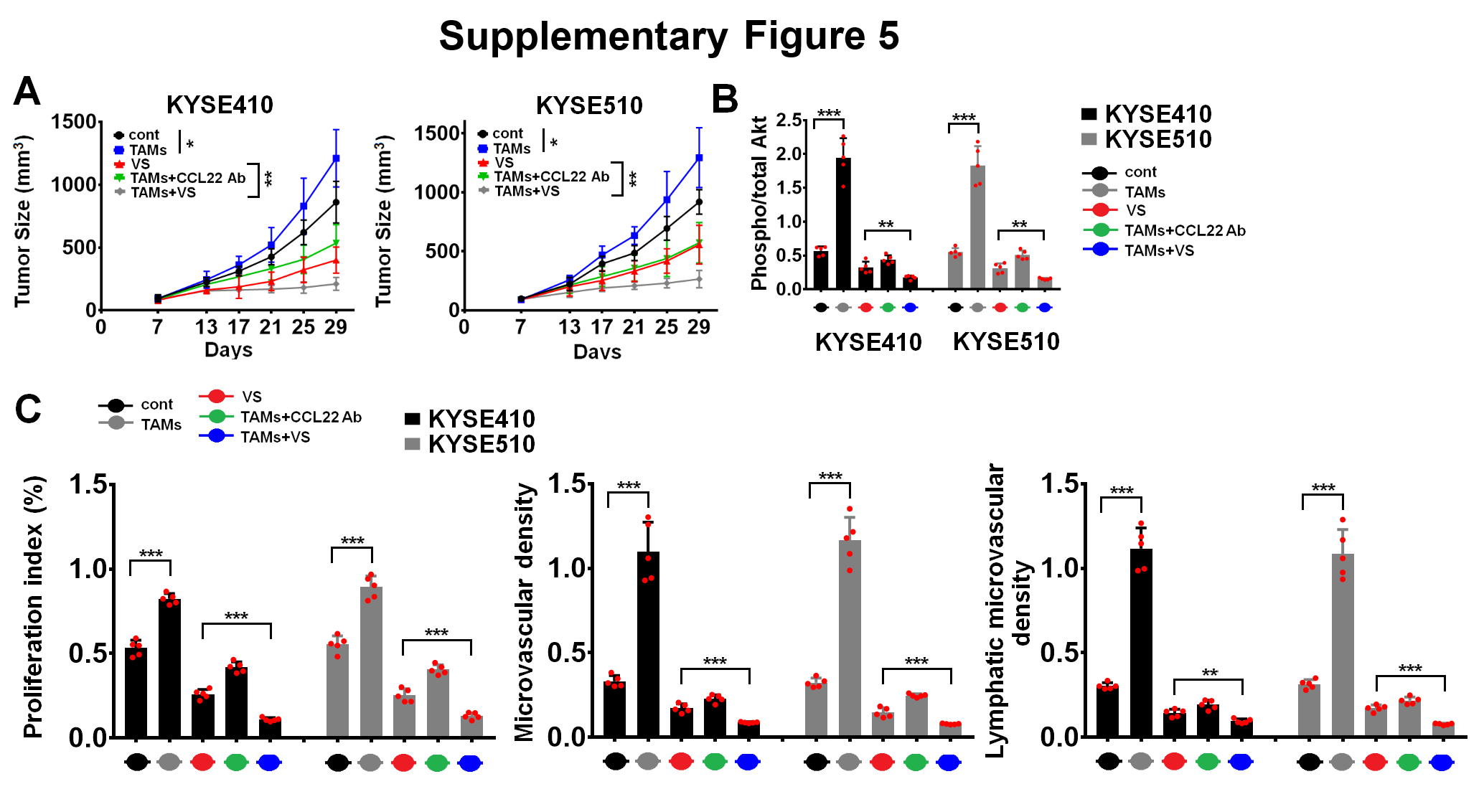


Supplementary Figure 5:

1. Mice bearing indicated ESCC tumors with pol-TAMs were treated with control solvent or CCL22 Ab (10 μg/mouse twice times per week, i.v.) or VS-6063 (25 mg/kg/day, p.o.). Growth curves of tumors were shown. (B) The ratio of pAKT/AKT in indicated ESCC tumors were evaluated using ELISA assay. (C) The expression of Ki-67 (proliferation index; left panel), CD31 (microvascular density; middle panel), or LYVE1 (lymphatic microvascular density; right panel) in indicated ESCC tumor tissues was evaluated using ELISA assay. * *P* < 0.05; ** *P* < 0.01; *** *P* < 0.001; two-tailed unpaired Student's *t*-test. Error bars, mean ± SD of five independent experiments.


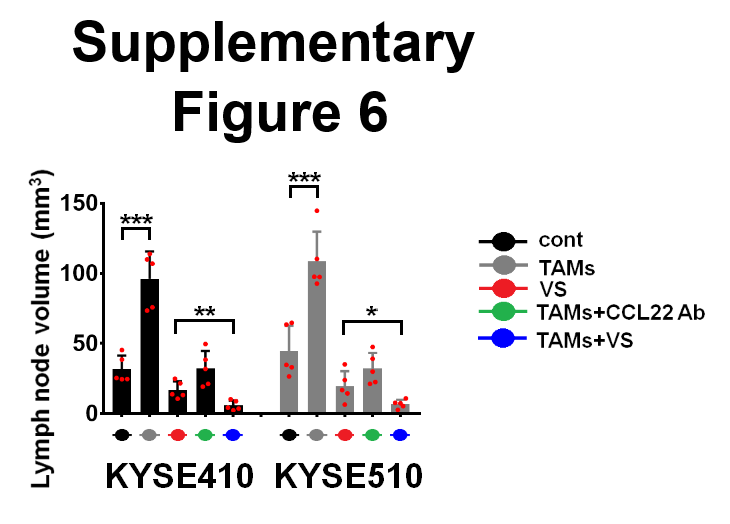


Supplementary Figure 6:

Foot pads of animals (n=5/group) were coinjected with KYSE410 or KYSE510 and pri-TAMs to establish the popliteal lymph node metastasis model and then evaluate the anti-lymph metastatic ability of VS-6063. The volume of lymph nodes was shown.


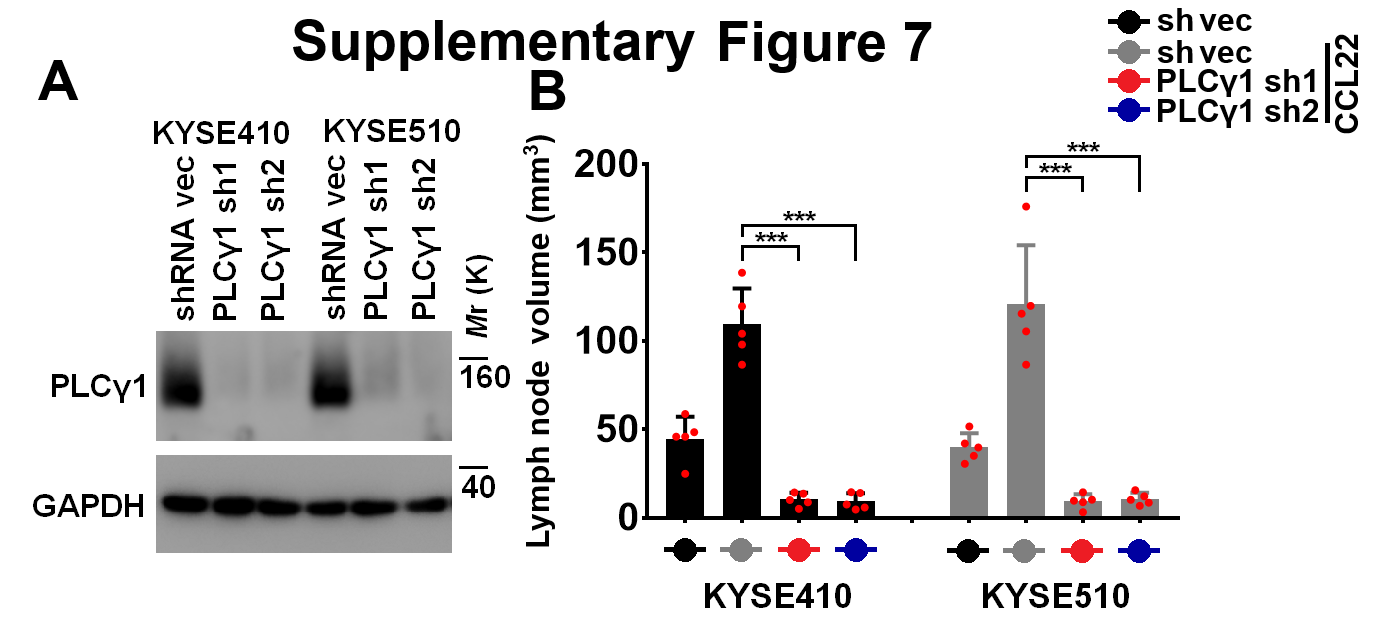


Supplementary Figure 7:

1. Stably silencing PLC-γ1 in 2 shRNA-transduced KYSE410 or KYSE510 cells analyzed by immunoblotting. GAPDH was used as a loading control. (B) Foot pads of animals (n=5/group) were coinjected with indicated KYSE410 or KYSE510 cells alone or intravenously injected rCCL22 at a dosage of 0.1 μg/kg biweekly alone or in the presence of VS-6063 (25 mg/kg/day, p.o.) to establish the popliteal lymph node metastasis model and then the volume of lymph nodes was shown. *** *P* < 0.001; two-tailed unpaired Student's *t*-test. Error bars, mean ± SD of five independent experiments.


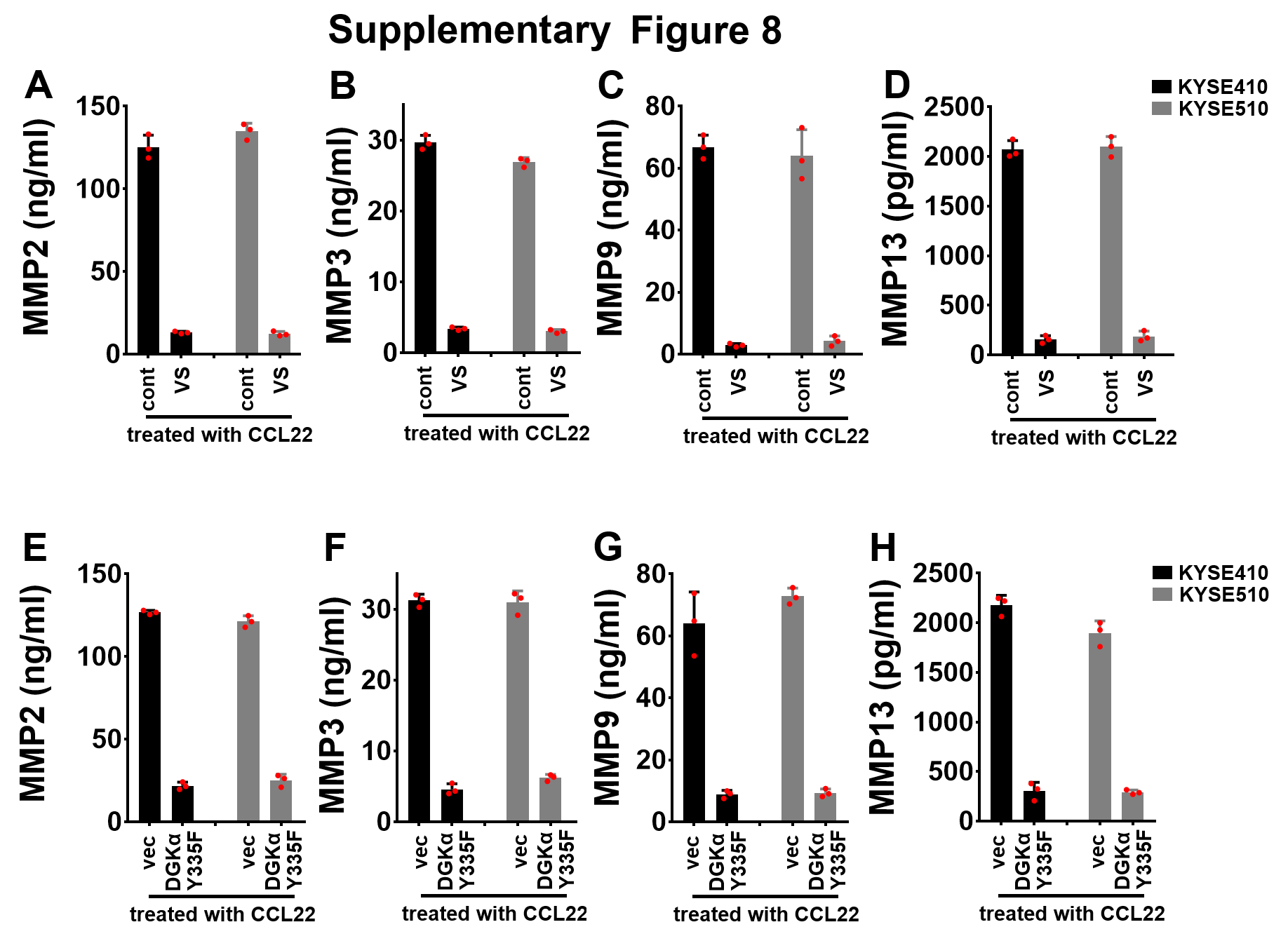


Supplementary Figure 8:

(A-D) KYSE410 or KYSE510 cells were cultured with CCL22 (50 ng/ml) for 5 days, and then treated with different doses of VS-6063 (2.5 μM). (E-H) KYSE410 or KYSE510 DGKα Y335F mutant cells were cultured with CCL22 (50 ng/ml) for 5 days. The secretion of MMP2, MMP3, MMP9, and MMP13 in the CM of indicated ESCC cells was evaluated using ELISA assay.
